# Evaluation of measurement properties of the German Work Role Functioning Questionnaire

**DOI:** 10.1186/s12889-022-13893-4

**Published:** 2022-09-15

**Authors:** Martina Michaelis, Monika A. Rieger, Stephanie Burgess, Viktoria Töws, Femke I. Abma, Ute Bültmann, Benjamin C. Amick, Eva Rothermund

**Affiliations:** 1 Institute of Occupational and Social Medicine and Health Services Research, University Hospital Tübingen, Wilhelmstr. 27, 72074 Tübingen, Germany; 2 Research Centre for Occupational and Social Medicine (FFAS), Freiburg, Germany; 3 Department of Psychosomatic Medicine and Psychotherapy, Ulm University Medical Center, Ulm, Germany; 4 Department of Health Sciences, University of Groningen, University Medical Center Groningen, Community and Occupational Medicine, Groningen, The Netherlands; 5 Fay W Boozman College of Public Health, University of Arkansas for Medical Sciences, Little Rock, AR USA; 6 Leadership Personality Centre Ulm (LPCU), Ulm University, Ulm, Germany

**Keywords:** Work capacity evaluation, Surveys and questionnaires, Adult, Psychometrics, Factor analysis, statistical

## Abstract

**Objective:**

We assessed the measurement properties of the German Work Role Functioning Questionnaire (WRFQ) after its cross-cultural adaptation of the Dutch version. The WRFQ is a generic role-specific instrument that measures how a particular health status influences the ability to meet work demands.

**Methods:**

We performed an observational study among German employees assessing the following measurement properties: 1) structural, 2) convergent and 3) discriminant validity, 4) floor and ceiling effects, 5) internal consistency, 6) reproducibility and 7) responsiveness. Participants were recruited from an online access panel sample aged 18 to 64 years having worked more than 12 hours in the last 4 weeks prior to study enrollment (n_(T0)_ = 653, n_(T1)_ = 66, n_(T2)_ = 95).

**Results:**

Measurement properties proved to be good except for structural validity and responsiveness. An exploratory factor analysis showed limited replicability of three of the four original subscales.

**Conclusion:**

With the WRFQ German version, the extent can be measured, to which employees with a certain health level experience problems can meet their work demands. This widely used health-related work outcome measurement tool, that helps to identify employees with decreasing work functioning, is now also available in German. This gives researchers and practitioners the opportunity to address work functioning in practice, e.g. in intervention studies in occupational health or rehabilitation. Further research to examine valid subscales is needed.

**Supplementary Information:**

The online version contains supplementary material available at 10.1186/s12889-022-13893-4.

## Introduction

The *Work Role Functioning Questionnaire* (WRFQ) is a generic role-specific instrument that measures the consequences of functional health status on the ability to accomplish work demands. Specifically, the WRFQ assesses the time (in percentage) in which workers experience difficulties in meeting work demands, such as work scheduling or physical demands, given their physical or emotional health status [1]. As a generic instrument, the WRFQ development was not restricted to a specific disease or occupation. Moreover, the instrument was developed to be used as work outcome measure in different research settings, such as health services, clinical trials, occupational health interventions, or rehabilitation.

The original American version of the WRFQ consists of 27 items and five subscales. The WRFQ has been cross-culturally adapted and was validated in Canadian French [2], Brazilian Portuguese [3], Dutch [4, 5], Spanish [6] and Norwegian and Danish [7]. During the cross-cultural adaptation to Dutch, a new version of the WRFQ 2.0 was developed which incorporates five new items covering additional working conditions encountered in current labor markets, and four scales, namely *Work scheduling and output demands, Physical demands, Mental and social demands* and *Flexibility demands* (WRFQ version 2.0 [4, 5, 7]; the respective items can be found in Additional file Table S1). After a cross-cultural adaptation from Dutch to German, we aim to present the measurement properties of the German WRFQ 2.0 version.

## Methods

### Cross-cultural adaptation into German

The adaptation of the Dutch WRFQ 2.0 into German followed the six-stage approach proposed by Beaton et al. [8]. The prefinal version was tested with a sample of 40 individuals (30 patients presenting psychosomatic symptoms, and 10 persons without symptoms), who also participated in cognitive interviews exploring issues such as content validity, wording, or logical structure of the items. Consequently, some items have been slightly adjusted to the German language usage.

Respondents were asked to assess the extent to which they have had difficulties meeting the work demands due to physical or mental health issues in the last 4 weeks (prior to completing the survey).

The 27 items were answered on a five point Likert scale ranging from 0 = difficult all of the time (calculated as 100%), 1 = difficult most of the time, 2 = difficult half of the time (50%), 3 = difficult some of the time, and 4 = difficult none of the time (0%). Each item also has the option ‘does not apply to my job’.

### Data analysis

The respective missing values generated by answering ‘does not apply to my job’ were imputed by the hot-deck algorithm in the program ‘r’ for the subsequent analyses.

For scale construction, the items were summed up with IBM SPSS 26, then divided by the number of items, followed by multiplication with 25 to obtain percentages between 0 (difficult all the time) and 100 (difficult none of the time). Thresholds of significance were set at *p* ≤ .05. Details of the cross-cultural adaption are part of a doctoral thesis [9].

### Design and sample

The sample was obtained from volunteers of a custom online panel (www.respondi.com) in Germany in 2018. Inclusion criteria for the online survey were aged 18–64, having worked more than 12 hours per week in the last 4 weeks prior to study participation and adequate reading comprehension skills in German. Excluded were individuals on parental leave, retirees, and self-employed. Participants received small monetary incentives (T0: 1.50 €, follow-ups: 1 €).

We targeted a sample size of about 600 respondents for the cross-sectional survey at T0, to have a sufficient number of employees in the subsequent multivariate subgroup analyses. This sample size was considered appropriate for the construct validation by following the rule of thumb of 10 cases per item of the WRFQ, i.e., *n* = 270, as recommended [10]. To conduct reproducibility and responsiveness analyses, two follow-up measurements were performed at 1 week (T1) and 3 months (T2) after the baseline measurement at T0. For the T1 and T2 follow-up, we targeted the participation of 50 and 100 individuals, respectively. For stable conditions we again controlled the inclusion and exclusion criteria mentioned above. The usability of the online survey was pretested among five employees.

Since the main purpose of the WRFQ is to measure the extent to which workers experience difficulties in meeting the work demands given a certain level of health, it was important to sample employees from different occupational settings. Therefore, an equiproportional quota sampling was defined based on the following three occupational categories: 1. blue-collar workers (e.g. workers in the manufacturing and processing industry, and craft professions), 2. gray-collar workers (e.g. health care, support and medical assistance occupations, service professions in the areas of facility management, caretakers, cleaning and security services, warehouse, and trade), and 3. white-collar workers (e.g., social workers, clerks and other respective professionals working in offices).

### Instrument validation

The investigation of the measurement properties of the German WRFQ followed the COSMIN-criteria [11], and consisted of the analysis of the structural, convergent and discriminant validity, floor and ceiling effects, internal consistency, reproducibility, and responsiveness. We aimed to replicate the Dutch validation study with no further development of the instrument. We therefore used the same methods of the working group of Abma et al. [5].

#### Structural validity

An exploratory factor analysis which was carried out by principal component analysis with eigenvalue criterion and varimax rotation. The factor structure was defined by taking into account items with loadings > 0.4 only [12].

#### Convergent and discriminant validity

The following constructs and instruments were used for the convergent validity analysis: productivity assessed with the *Endicott Work Productivity Scale* (EWPS [13];), overall work ability with the single item derived from the *Work Ability Index* (WAI; ‘Assuming that the highest work ability you have ever had is 10, how would you rate your current work ability?’, 0 = absolutely unable to work to 10 = best work ability [14]), *Decision latitude* and *Job demands* with the *Job Content Questionnaire* (JCQ [15]), and *General health* with the respective single item derived from the 12-item *Short Form Survey of General Health* (SF-12) health questionnaire [16]. Convergent validity was determined by assessing the extent to which the strength of the correlations (Pearson or Spearman rho) of the WRFQ with similar constructs agrees with a set of pre-defined hypotheses. High discriminant validity was expected by detecting low correlations with non-related constructs. Correlations were classified as either small (0.15 ≤ r < 0.25), moderate (0.25 ≤ r < 0.35), or large (0.35 ≤ r) [17].

The hypotheses of the convergent validity (no. H1–3) and discriminant validity (no. H4 and H5) analyses were: A high WRFQ total scale value correlates …

- H1: … with a high work productivity value (*EWPS* scale; moderately to highly).
- H2: … with a good self-reported general health value (SF-12 item *General health*) (moderately).
- H3: … with a good overall work ability (*WAI* item) (moderately).
- H4: … with a high decision latitude (*JCQ* subscale; lowly).
- H5: … with low psychological job demands (*JCQ* subscale; lowly).

Both convergent and discriminant validity measured by the correlation coefficient of Spearman are considered acceptable if at least 75% of the hypotheses are confirmed [10].

#### Floor and ceiling effects of scales

Floor and ceiling effects of a scale were considered present if more than 15% of the responses were at the lowest or highest attainable scores of the scale, respectively [10]).

#### Internal consistency

The reliability of the items was analyzed assessing Cronbach’s α, the intraclass correlation coefficient (ICC), and the inter-item and item-to-total correlations of the scales. Cronbach’s α and ICC greater than 0.7 are considered appropriate for group comparisons [18]. Inter-item and item-to-total correlations were considered appropriate if they were included in the intervals 0.2 and 0.8, and 0.3 and 0.9, respectively [19].

#### Reproducibility

The reproducibility was assessed with the ICC, and was considered acceptable at the group and individual level for ICC > 0.7 and ICC > 0.9, respectively [18]). Additionally, the standard error of measurement (SEM) was calculated by SD_diff_/√2.

#### Responsiveness

The sensitivity of the instrument to measure changes between T0 and T2 was evaluated by comparing the mean changes of the WRFQ and of the overall work ability (global item). In addition, the responses to two additional items at T2, the so-called global perceived effect (GPE) items, which measure the extent to which respondents perceived changes in their mental and physical work ability since baseline (e.g., ‘to what extent has your work ability changed regarding the mental demands at work in the last 3 months?’, 1 = much better, 5 = much worse) were examined.

The mean change of the WRFQ scores was estimated for the total scale and subscales by calculating the mean differences between T0 und and T2 and the respective standard deviations (SDs). The standardized response mean (SRM; ratio between the mean change score and its SD) was calculated for all scores (WRFQ total and subscales). Furthermore, the WRFQ mean changes were correlated with mean changes of work ability and the respective GPE items by Spearman correlation coefficient rho.

SRM effect size categories were defined as < 0.2 (trivial), ≥0.2- < 0.5 (small), ≥0.5- < 0.8 (moderate) and ≥ .80 (large) [20]. An at least moderate correlation between the WRFQ measurement change and the change of work ability between T0 and T2 was expected, as well as stable responses in a large part of the sample. On the basis of this set of change measures, the following hypothesis was formulated:

- Hypothesis H6: a) The correlation of the changes in overall work ability and the GPE items on mental and physical work ability is high. The correlation between the WRFQ mean change scores and b) the global perceived effect (GPE) items of work ability and c) the change of the global work ability item between T0 and T2 are at least moderate.

## Results

### Response rate and sample

At T0, 4.694 participants of the online access panel were addressed. The final sample consisted of 653 employees (response rate 14%; see Additional file Table S2). The sample sizes and response rates at T1 and T2 follow-up were n_T1_ = 66 (33%), and n_T2_ = 95 (16%), respectively. No major differences were found concerning age, gender and job type between the T0 and T2 samples.

The respondents at T0 consisted of 239 white-, 194 Gy- and 220 blue-collar workers (36.6, 29.7 and 33.7%, respectively). Nearly half of the sample was female (47.3%); the average age was 43 ± 12 years. Almost a quarter (24.0%) had jobs with shift work and 60.3% participants worked in small or medium-sized companies. Almost two thirds (58.8%) reported excellent/good health and rated their global work ability on average at 8.6 (SD 1.8, range 0–10) (see Additional file Table S3).

### Descriptive results of the WRFQ items

Item means ranged from 2.4 (SD 1.2; no. 9 ‘Feel a sense of accomplishment in your work’) to 3.6 (SD 0.8; no. 15 ‘Use hand-held tools or equipment’) (see Additional file Table S4). The option ‘Does not apply to my job’ was answered between 6.0 and 20.4% for the following five items: ‘Lift, carry, or move objects at work weighing more than 5 kg’, ‘Use hand-held tools or equipment’, ‘Ability to concentrate for reading and processing the information’, ‘Speak with people in-person’, and ‘Process incoming information’ (items no. 11, 15, 20, 21, and 25).

### Structural validity

The exploratory factor analysis revealed a factor structure based on four subscales, but the factor content of the German version was different from the Dutch version. The subscales *Mental and social demands* and *Flexibility demands* described in the Dutch version were identified as one subscale in the German data (Factor 1). The Dutch subscale *Work scheduling and output demands*, on the other hand, was divided into two subscales in the German data (Factor 2 and 4). The subscale *Physical demands* could be well replicated (Factor 3) (see Table 1).

**Table 1:** Factor structure (German version) at T0 vs. factor structure in a Dutch sample reported by Abma et al. [5]

Factor no. (German sample)	Item	Item no.	FAC1	FAC2	FAC3	FAC4	Factor (Dutch sample) ^a**)**^
Factor 1	Use hand-held tools or equipment ^b)^	15	**0.557**		0.434		F3
	Keep your mind on your work	16	**0.618**			0.536	F2
	Do work carefully	17	**0.691**				F2
	Concentrate on your work	18	**0.654**			0.558	F2
	Easily read or use your eyes when working	20	**0.656**				F2
	Speak with people in-person, in meetings or on the phone	21	**0.648**				F2
	Control your temper around people when working	22	**0.511**				F2
	Set priorities in my work	23	**0.725**				F4
	Handle changes in my work	24	**0.685**				F4
	Process incoming information, for example e-mails, in time	25	**0.693**				F4
	Perform multiple tasks at the same time	26	**0.675**				F4
	Be proactive, show initiative in my work	27	**0.615**				
Factor 2	Start on your job as soon as you arrived at work	2		**0.625**		0.449	F1
	Stick to a routine or schedule	4		**0.779**			F1
	Work fast enough	5		**0.793**			F1
	Finish work on time	6		**0.846**			F1
	Do your work without making mistakes	7		**0.758**			F1
Factor 3	Feel you have done what you are capable of doing	10			**0.404**		F1
	Lift, carry, or move objects at work weighing more than 10 pounds	11			**0.689**		F3
	Sit, stand, or stay in one position for longer than 15 min while working	12			**0.734**		F3
	Repeat the same motions over and over again while working	13			**0.771**		F3
	Bend, twist, or reach while working	14			**0.676**		F3
Factor 4	Get going easily at the beginning of the workday	1				**0.602**	F1
	Do your work without stopping to take extra breaks or rests	3		0.475		**0.488**	F1
	Feel a sense of accomplishment in your work	9				**0.565**	F1
	Work without losing your train of thought	19	0.538			**0.591**	F2
–	Satisfy the people who judge your work	8					F1

*Abbreviation*: *FAC* Factor loading at factors 1 to 4

^a^ Abma et al. [5]; factors F1 = Work scheduling and output demands, F2 = Mental and social demands, F3 = Physical demands, F4 = Flexibility demands

^b^ For example, a phone, pen, keyboard, computer mouse, drill, hairdryer or sander

Comment: Factor loading ≤ .4 not shown. Methods: Extraction method = principal component analysis; rotation method = varimax with Kaiser normalization. Total variance explained = 59.1%

In close reference to the Dutch version, the four subscales derived from the factor analysis were named as follows: WRFQ-F1 *Work scheduling and output demands* (10 items), WRFQ-F2 *Physical demands* (5 items), WRFQ-F3 *Mental and social demands* (7 items), and WRFQ-F4 *Flexibility demands* (5 items).

Table 2 shows the results of the convergent and discriminant validity analysis. In agreement with hypotheses H1 to H3 (convergent validity), the correlations of the WRFQ total scale and subscales with the EWPS productivity, the SF-12 global health item, and the global work ability item (WAI) were moderately to large. Also the discriminant validity assumed in H4 and H5 (*Decision latitude* and *Psychological job demands*) could be confirmed.

**Table 2:** Correlation results (convergent and discriminant validity; Spearmans’ rho; T0; *n* = 653)

		Subscale dimensions			
Variables/scales	WRFQ_**(total)**_	WRFQ-F1	WRFQ-F2	WRFQ-F3	WRFQ-F4
WRFQ_(total)_		0.88	0.67	0.75	0.72
WRFQ-F1 (Work scheduling and output demands)			0.45	0.51	0.53
WRFQ-F2 (Physical demands)				0.37	0.32
WRFQ-F3 (Mental and social demands)					0.67
WRFQ-F4 (Flexibility demands)					
**Correlation between WRFQ and other constructs**					
Endicott Work Productivity Scale (EWPS), *hypothesis 1*	−0.49	−0.46	−0.21	−0.47	−0.44
General health (SF-12 global item), *hypothesis 2*	−0.33	−0.27	−0.36	−0.29	−0.23
Work Ability Index (WAI, global item), *hypothesis 3*	0.40	0.27	0.22	0.37	0.34
Work Ability Index (WAI, physical demands)	−0.38	− 0.31	− 0.35	−0.30	− 0.30
Work Ability Index (WAI, mental demands)	−0.44	− 0.42	−0.25	− 0.37	−0.37
Decision latitude (JCQ), *hypothesis 4*	0.07	−0.01	0.03	0.18	0.11
Psychological job demands (JCQ), *hypothesis 5*	−0.16	−0.14	− 0.15	−0.15	− 0.13

*Legend: P*-values: Always *p* ≤ .001 with the exception of correlation between Subscale *Decision latitude* (Job Content Quest; JCQ) and WRFQ total score / subscore F1 / subscore F2 (*p* = .057/0.829/0.447) as well WRFQ total score / subscore 4 (*p* = .911/0.304)

### Floor/ceiling effects, internal consistency and reproducibility

Neither floor nor ceiling effects were detected (Table 3, see columns entitled with T0). The highest proportion reaching the highest attainable scale value of 100 was found for *Flexibility demands* with 13.5%.

**Table 3:** Psychometric properties of the German version of the WRFQ and its subscales

	T0 (***n*** = 653)							T0-T1 (***n*** = 66)			T0-T2 (***n*** = 96)		
Scale/ subscale	Mean (SD) ^a**)**^	N (%) at floor (0%)	N (%) at ceiling (100%)	Cronbach’s α	ICC (CI_**95%**_)	Inter-item correlation (mean (SD))	Item-to-total correlation	Mean (SD) T0/T1	ICC	SEM	Mean (SD) T0/T2	Mean change T0-T2 (SD)	SRM
**WRFQ**_**(total)**_	71.2 (17.4)	0 (0%)	0 (0%)	0.94	0.94 (0.93–0.95)	0.37 (0.14)	0.43–0.73	69.8 (17.5)/ 77.3 (16.6)	0.80	0.63	72.6 (15.5)/ 54.6 (13.0)	−17.96 (13.36)	1.34
WRFQ-F1 Work scheduling and output demands (10 items)	68.7 (21.8)	0 (0%)	16 (2.5%)	0.89	0.89 (0.88–0.90)	0.44 (0.18)	0.48–0.76	68.2 (20.7)/ 73.7 (19.8)	0.77	0.63	71.2 (20.3)/ 50.3 (16.7)	−20.86 (17.62)	1.18
WRFQ-F2 Physical demands (5 items)	69.2 (22.8)	1 (0.2%)	83 (12.7%)	0.79	0.78 (0.76–0.81)	0.43 (0.009)	0.45–0.65	65.8 (23.0)/ 77.3 (19.7)	0.44	2.38	66.8 (23.8)/ 53.1 (18.6)	−18.05 (23.42)	0.77
WRFQ-F3 Mental and social demands (7 items)	74.0 (19.8)	1 (0.2%)	41 (6.3%)	0.87	0.87 (0.86–89)	0.50 (0.02)	0.46–0.81	72.9 (21.2)/ 78.5 (18.9)	0.67	1.62	75.9 (18.1)/ 58.2 (15.2)	−12.95 (20.52)	0.63
WRFQ-F4 Flexibility demands (5 items)	75.0 (21.3)	6 (0.9%)	88 (13.5%)	0.85	0.85 (0.83–0.87)	0.53 (0.003)	0.59–0.70	74.7 (23.4)/ 82.6 (20.6)	0.65	1.95	77.5 (19.9)/ 59.8 (16.7)	−11.33 (23.25)	0.49

*Abbreviations*: *CI* Confidence interval, *ICC* Intraclass coefficient, *SD* Standard deviation, *SEM* Standard error of measurement (SD_diff_/√2), *SRM* Standardized response mean (ratio between mean change score and its SD)

Floor and ceiling effects and internal consistency (T0), reproducibility by reliability and test-retest reliability (T1) and responsiveness (T2)

^a^ Range 0–100 (lowest to best work role functioning) in all scales

The internal consistency was appropriate with Cronbach’s α. The ICC estimates were equal or above the threshold of 0.7. Moreover, the values for the inter-item (between 0.2 and 0.8) and item-to-total correlations (between 0.3 and 0.9) affirmed the internal consistency of the German WRFQ (see again Table 3, T0).

The reproducibility of the instrument at T1 was acceptable at the group level with ICC > 0.8.

### Responsiveness

Means of WRFQ values at T0 and T2 and mean change scores are also reported in Table 3. The change of the total WRFQ score was − 17.96 (SD 13.36). The highest change was found for the subscale Work *scheduling and output demands*, followed by *Physical demands* and *Mental and social demands* with lower decreases. The values indicate a reduced work function with high effect sizes at T2 (SRM = 1.34 for the total WRFQ score).

The overall assessed current work ability value deteriorated from 8.7 to 8.0 between T0 at T2. The mean change was − 0.63 (SD 1.7), indicating a small difference (SRM = 0.37).

To answer hypothesis H6, we found a weak correlation between the mean change scores of WRFQ and the work ability item between T0 and T1 (rho = 0.19; see Additional file Table S5). This effect was supported by lacking correlations with the subjective assessments of the respondents, namely the GPE items concerning subjective changes in physical and mental work ability (rho ≤0.09 and 0.13), with the exception of a small correlation with subscale *Mental and social demands*; rho =0.18 and 0.20, respectively).

## Discussion

We evaluated the measurement properties of the cross-culturally adapted from the further developed Dutch version of the Work Role Functioning Questionnaire in a German working population. The translated and adapted instrument shows good structural validity, although the subscales were only replicable to a limited extent compared to the Dutch version. The total WRFQ scale, however, can be seen as an international comparable instrument.

Since the subscale *Flexibility demands* of the Dutch version could not be replicated in the present study, it seems that there is some semantic overlap between items 16 to 22 (*Mental and social demands*) and items 23 to 27 (*Flexibility demands*) in the Dutch version. This might lead respondents to a similar reference frame of interpretation. In addition, this seems to be supported by the fact that the highest subscale correlation with r = 0.77 in the Dutch version were observed between the subscales *Mental and social demands* and *Flexibility demands* [7].

Most of the other measurement properties of the German version of WRFQ (internal consistency, reproducibility and floor or ceiling effects) were good. The moderately large correlations between the WRFQ score and *Work productivity* (*EWPS*), the *Overall work ability and General health* items, and the lacking correlations with the two *JCQ* scales indicate that the WRFQ and those constructs measure related, but not the same construct. Hence, there was evidence of convergent and discriminant validity of the German WRFQ.

The responsiveness of the instrument was not sufficient. This goes in line with previous results of a Dutch and a Spanish working population, which showed only moderate responsiveness [5, 6]. The relatively large mean change of the WRFQ score between T0 and T2 might indicate a significant self-selection mechanism to the T2 sample.

Given the lack of WAI differences between T0 and T2, we do not assume major health deterioration and associated work role functioning reduction in the T2 population at 3 months. However, we cannot state it precisely, as we did not repeat the question of global health at T2. This must be regarded as a study limitation and implicates further research.

### Strength and limitations of the study

The major strength of our study is the validation of a generic instrument to assess the health-related work functioning of working people in view of the common and important aspects such as work scheduling and physical, mental as well as social demands at work. A further strength is the validation of an instrument on a working general population sample that was originally developed for people with health problems. This has so far only been done in the Netherlands, Norway and Denmark. A further strength is the responsiveness test of the instrument, which has not often been tested and is an addition to the literature.

Limitations are the restricted representativeness of members of an online access panel for the working population: Not all professions and occupational positions could be mapped in our sample. Online access panels are generally limited by the typically low number of individuals in management positions for example. Thus, further studies are advantageous in these subgroups. A critical discussion of online access panels can be found in Burgess et al. [21]. The restricted knowledge about the health status of the sample is a further limitation.

## Conclusions

The German WRFQ is a short, psychometrically valid instrument consisting of 27 items. It can be used in the assessment and monitoring of work functioning of workers of different ages, with different health status and occupations. The WRFQ may be used as a work outcome parameter in interventions aiming at maintaining the functioning at work or employability after return to work.

## Supplementary Information

**Additional file 1: Table S1.** Dimensions and items of the WRFQ 2.0 (Dutch version) with 27 items.

**Additional file 2: Table S2.** Number of participants and response rates at different survey times.

**Additional file 3: Table S3.** Sample description.

**Additional file 4: Table S4.** Descriptive results of 27 German WRFQ items.

**Additional file 5: Table S5.** Correlation between German WRFQ mean change scores and change of work ability.

## Data Availability

The datasets analyzed during the current study are available from the corresponding author on reasonable request.

## Abbreviations

EWPS: Endicott Work Productivity Scale
GPE: Global perceived effect
ICC: Intraclass correlation coefficient
JCQ: Job Content Questionnaire
SD: Standard deviation
SEM: Standard error of measurement
SF-12: Short Form Survey of General Health (12-items)
SRM: Standardized response mean
WAI: Work Ability Index
WRFQ: Work Role Functioning Questionnaire
