# Supplementary material for: Evaluation of measurement properties of the German Work Role Functioning Questionnaire
Source: BMC Public Health. 2022 Sep 15;22:1750. doi: 10.1186/s12889-022-13893-4 (PMC9479368; doi:10.1186/s12889-022-13893-4)
Supplement: Supplementary file 1 — Additional file 1: Table S1. Dimensions and items of the WRFQ 2.0 (Dutch version) with 27 items. [file 12889_2022_13893_MOESM1_ESM.pdf]

Table S1 Dimensions and items of the WRFQ 2.0 (Dutch version) with 27 items

Dimensions	Items
Work scheduling and output demands (10 items)	Get going easily at the beginning of the workday
	Start on your job as soon as you arrived at work
	Do your work without stopping to take extra breaks or rests
	Stick to a routine or schedule
	Work fast enough
	Finish work on time
	Do your work without making mistakes
	Satisfy the people who judge your work
	Feel a sense of accomplishment in your work
	Feel you have done what you are capable of doing
Physical demands (5 items)	Lift, carry, or move objects at work weighing more than 10 pounds
	Sit, stand, or stay in one position for longer than 15 min while working
	Repeat the same motions over and over again while working
	Bend, twist, or reach while working
	Use hand-held tools or equipment (for example, a phone, pen, keyboard, computer mouse, drill, hairdryer or sander)
Mental and social demands (7 items)	Keep your mind on your work
	Do work carefully
	Concentrate on your work
	Work without losing your train of thought
	Easily read or use your eyes when working
	Speak with people in-person, in meetings or on the phone
	Control your temper around people when working
Flexibility demands (5 items)	Set priorities in my work (new)
	Handle changes in my work (new)
	Process incoming information, for example e-mails, in time (new)
	Perform multiple tasks at the same time (new)
	Be proactive, show initiative in my work (new)
