# Supplementary material for: Evaluation of measurement properties of the German Work Role Functioning Questionnaire
Source: BMC Public Health. 2022 Sep 15;22:1750. doi: 10.1186/s12889-022-13893-4 (PMC9479368; doi:10.1186/s12889-022-13893-4)
Supplement: Supplementary file 2 — Additional file 2: Table S2. Number of participants and response rates at different survey times. [file 12889_2022_13893_MOESM2_ESM.pdf]

Table S2 Number of participants and response rates at different survey times

	T0		T1		T2	
**Time of survey**	**n**	**%**	**n**	**%**	**n**	**%**
Sample size addressed	4694		197		581	
Non-responder	2616	55.7	112	56.9	461	79.3
Drop-out	230	4.9	3	1.5	4	0.7
Quota full	585	12.5	0	0.0	0	0.0
Removed due to quality reasons	604	12.9	15	7.6	21	3.6
Missing value of job type	6	0.1	1	0.5	0	0.0
**Data sets analyzable**	**653**	**13.9**	**66**	**33.5**	**95**	**16.4**

Legend

T0= baseline, T1= one week after T0, T2= three months after T0
