# Supplementary material for: Evaluation of measurement properties of the German Work Role Functioning Questionnaire
Source: BMC Public Health. 2022 Sep 15;22:1750. doi: 10.1186/s12889-022-13893-4 (PMC9479368; doi:10.1186/s12889-022-13893-4)
Supplement: Supplementary file 3 — Additional file 3: Table S3. Sample description. [file 12889_2022_13893_MOESM3_ESM.pdf]

Table S3 Sample description (T0; raw data, n=653)

Aspect			Gender				Job type … -collar workers					
	Total (n=647)		Female (n=306, 47.3%)		Male (n=341, 52.7%)		White (n=239, 36.6%)		Gray (n=194, 29.5%)		Blue (n=220, 33.8%)	
	N or mean	{SD} or %)	N or mean	{SD} or %)	N or mean	{SD} or %)	N or mean	{SD} or %)	N or mean	{SD} or %)	N or mean	{SD} or %)
Age (years), mean {SD}	43.1	{11.8}	41.8	{12.0}	44.2	{11.5}	42.3	{11.7}	43.2	{11.7}	43.9	33.3%
Gender (female), N (%)	306	47.0%					155	65.4%	105	55.0%	46	21.0%
Education												
No graduation	5	0.8%	3	1.0%	2	0.6%	1	0.4%	4	2.1%	0	0.0%
Primary school	107	16.5%	33	10.8%	74	21.7%	10	4.2%	37	19.4%	60	27.4%
Secondary school	377	58.3%	184	60.1%	193	56.6%	121	51.1%	113	59.2%	143	65.3%
High school	158	24.4%	86	28.1%	72	21.1%	105	44.3%	37	19.4%	16	7.3%
Job tenure (years)	23.7	{12.5}	21.8	{12.5}	25.4	{12.3}	21.5	{12.2}	23.8	{12.5}	26.1	{12.4}
Shift work (yes)	155	24.0%	69	22.5%	86	25.2%	14	5.9%	68	35.7%	73	{11.9}
Working hours/week	34.6	{9.3}	36.1	{8.2}	32.8	{10.2}	34.3	{9.2}	32.4	{11.3}	36.7	{6.6}
Company size (No. of employees)												
Small (<10)	102	15.6%	52	8.0%	50	7.7%	28	4.3%	31	4.7%	43	6.6%
Medium (<250)	292	44.7%	134	20.5%	158	24.2%	102	15.6%	87	13.3%	99	15.2%
Large (>250)	259	39.7%	467	71.5%	445	68.1%	523	80.1%	535	82.0%	511	78.2%
EWPS Endicott work productivity	16.0	{12.8}	16.9	{12.5}	15.1	{12.9}	18.2	{13.4}	14.9	{4.6}	14.5	{12.4}
JCQ-DL Decision latitude	65.7	{9.7}	65.0	{10.3}	66.4	{9.1}	65.5	{9.5}	65.8	{10.1}	65.9 {9.	5}
JCQ_PD Psychological job demands	27.3	{5.0}	26.8	{5.1}	27.7	{4.9}	26.0	{4.7}	27.8	{5.2}	28.2	{4.8}
SF-12 overall item												
Excellent	107	16.4%	52	16.9%	55	15.9%	46	19.4%	24	12.6%	35	16.0%
Good	277	42.4%	120	39.1%	157	45.4%	101	42.6%	78	40.8%	95	43.4%
Satisfactory	172	26.3%	72	23.5%	100	28.9%	52	21.9%	59	30.9%	60	27.4%
Fair	87	13.3%	62	20.2%	25	7.2%	37	15.6%	28	14.7%	22	10.0%
Poor	10	1.5%	1	0.3%	9	2.6%	1	0.4%	2	1.0%	7	3.2%
SF-12-PCS (physical) (1)	50.1	{8.5}	49.7	{8.9}	50.4	{8.1}	51.6	{8.4}	49.2	{8.2}	49.3	{8.7}
SF-12-MCS (mental)	48.0	{11.6}	46.1	{12.2}	49.8	{10.7}	46.5	{12.1}	48.3	{11.2}	49.4	{11.1}
WAI (global) (2)	8.6	{1.8}	8.4	{1.8}	8.7	{1.7}	8.3	{1.9}	8.7	{1.8}	8.7	{0.9}
WAI (physical) (2)	2.0	{0.9}	2.1	{0.9}	2.0	{0.9}	2.0	{0.9}	2.1	{0.9}	2.0	{1.7}
WAI (mental) (2)	2.2	{1.0}	2.3	{1.0}	2.0	{0.9}	2.3	{1.0}	2.1	{0.9}	2.0	{10.5}
WAI (in two years from now) (2)	2.7	{0.5}	2.7	{0.5}	2.7	{0.5}	2.7	{0.4}	2.6	{0.5}	2.7	{10.6}
Job satisfaction overall item (1)	2.0	{0.6}	2.0	{0.6}	2.0	{0.6}	2.0	{0.6}	2.0	{0.6}	2.0	{0.6}

Legend

Abbreviations: JCQ = Job Content Questionnaire; MCS = Mental Component Summary; SD: standard deviation; SF12 General Health Scale; PCS = Physical Component Summary; WAI = Work Ability Index

(1) Classified according to the German SOEP (Socio-Economic Panel)

(2) Single item, ranges: global: from 1 = unable to work to 11 = best ability to work; physical: from 1 = very good to 5 = very poor; mental: from 1 = very good to 5 = very poor; work ability in two years from now: from 1 = unlikely to 3 = fairly sure; job satisfaction: from 1 = very satisfied to 4 = very dissatisfied

EWPS, ranges: from 0 = best score to 100 = worst score; JCQ-DL, ranges from 1 = absolutely not right to 4 = absolutely right (high values = high demands); JCQ_PD, ranges from 1 = absolutely not right to 4 = absolutely right (high values = high decision latitude); SF12, ranges from 0 = worst score value to 100 = best score value (scale mean = 50, SD = 10)
