# Supplementary material for: Evaluation of measurement properties of the German Work Role Functioning Questionnaire
Source: BMC Public Health. 2022 Sep 15;22:1750. doi: 10.1186/s12889-022-13893-4 (PMC9479368; doi:10.1186/s12889-022-13893-4)
Supplement: Supplementary file 4 — Additional file 4: Table S4. Descriptive results of 27 German WRFQ items. [file 12889_2022_13893_MOESM4_ESM.pdf]

Table S4 Descriptive results of 27 German WRFQ items (T0)

Mean values [1] and standard deviations of imputed data; n = 653 and number of missing values in raw data

		Imputed data		Raw data	
**No.**	**Item**	**Mean**	**SD**	**$N_{(valid)}$**	**Missing values (%) [2]**
1	Get going easily at the beginning of the workday	2.61	1.11	647	0.9
2	Start on your job as soon as you arrived at work	2.82	1.23	641	1.8
3	Do your work without stopping to take extra breaks or rests	2.63	1.21	634	2.9
4	Stick to a routine or schedule	2.94	1.25	633	3.1
5	Work fast enough	2.88	1.20	634	2.9
6	Finish work on time	2.96	1.27	632	3.2
7	Do your work without making mistakes	2.94	1.24	646	1.1
8	Satisfy the people who judge your work	3.21	1.01	635	2.8
9	Feel a sense of accomplishment in your work	2.41	1.24	650	0.5
10	Feel you have done what you are capable of doing	2.62	1.31	645	1.2
11	Lift, carry, or move objects at work weighing more than 10 pounds	2.81	1.30	520	20.4
12	Sit, stand, or stay in one position for longer than 15 min while working	2.90	1.31	630	3.5
13	Repeat the same motions over and over again while working	2.82	1.24	572	12.4
14	Bend, twist, or reach while working	3.08	1.04	616	5.7
15	Use hand-held tools or equipment [3]	3.57	0.84	614	6.0
16	Keep your mind on your work	3.09	0.97	651	0.3
17	Do work carefully	3.26	1.03	651	0.3
18	Concentrate on your work	3.04	1.02	652	0.2
19	Work without losing your train of thought	2.82	0.98	651	0.3
20	Easily read or use your eyes when working	3.08	0.97	578	11.5
21	Speak with people in-person. in meetings or on the phone	3.34	0.93	551	15.6
22	Control your temper around people when working	3.07	1.05	632	3.2
23	Set priorities in my work	3.15	0.99	647	0.9
24	Handle changes in my work	3.09	1.06	642	1.7
25	Process incoming information, for example e-mails, in time	3.14	1.06	552	15.5
26	Performing multiple tasks at the same time	2.96	1.07	630	3.5
27	Being proactive, taking initiative at work	3.27	1.06	644	1.4

Legend
Abbreviations: SD= standard deviation.
[1] Value ranges on a 5-point Likert scale from 0 = difficult all the time (100%), 1 = most of the time, 2 = half of the time
[2] Missing data: "Does not apply to my job"
[3] For example, a phone, pen, keyboard, computer mouse, drill, hairdryer or sander
