# Supplementary material for: Evaluation of measurement properties of the German Work Role Functioning Questionnaire
Source: BMC Public Health. 2022 Sep 15;22:1750. doi: 10.1186/s12889-022-13893-4 (PMC9479368; doi:10.1186/s12889-022-13893-4)
Supplement: Supplementary file 5 — Additional file 5: Table S5. Correlation between German WRFQ mean change scores and change of work ability. [file 12889_2022_13893_MOESM5_ESM.pdf]

Table S5 Correlation between German WRFQ mean change scores and change of work ability (n=95)

a. Global Perceived Effect items (GPE) and

b. mean change of global Work Ability Index (WAI) item between T0 and T2 after 3 month

**Current Work Ability (WAI):**	**a. Global Perceived Effect (GPE) items at T2**				**b. Global WAI item**	
	**Physical**		**Mental**		**Mean change value (T0 - T2)**	
**WRFQ mean change scores**	**rho**	**p**	**rho**	**p**	**rho**	**p**
$WRFQ_{(total)}$	0.09	.402	0.13	.213	0.19	.057
WRFQ-F1 Work scheduling and output demands	-0.06	.548	0.00	.965	0.15	.157
WRFQ-F2 Physical demands	0.04	.713	0.04	.725	0.15	.149
WRFQ-F3 Mental and social demands	0.20	.048	0.18	.072	0.16	.117
WRFQ-F4 Flexibility demands	0.06	.532	0.10	.326	0.18	.075

Legend
GPE: 'To what extent has your work ability changed regarding the physical/mental demands at work in the last three months?' from 1 = much better to 5 = much worse
WAI: 'Assuming that the highest work ability you have ever had is 10, how would you rate your current work ability?' from 0 = absolutely unable to work to 10 = best work ability
Abbreviations: p = significance value, rho = Spearman correlation coefficient
